# Glial remodeling and choroidal vascular pathology in eyes from two donors with Choroideremia

**DOI:** 10.3389/fopht.2022.994566

**Published:** 2022-10-14

**Authors:** Malia M. Edwards, D. Scott McLeod, Rhonda Grebe, Imran A. Bhutto, Richa Dahake, Kelly Crumley, Gerard A. Lutty

**Affiliations:** The Wilmer Eye Institute, Johns Hopkins University School of Medicine, Baltimore, MD, United States

**Keywords:** Choroideremia, Müller cells, glia, Choroid, choriocapillaris, remodeling

## Abstract

Choroideremia (CHM) is a recessive, X-linked disease that affects 1 in 50,000 people worldwide. CHM causes night blindness in teenage years with vision loss progressing over the next two to three decades. While CHM is known to cause progressive loss of retinal pigment epithelial (RPE) cells, photoreceptors and choroidal vessels, little attention has been given to retinal glial changes in eyes with CHM. In addition, while choroidal loss has been observed clinically, no histopathologic assessment of choroidal loss has been done. We investigated glial remodeling and activation as well as choriocapillaris changes and their association with RPE loss in postmortem eyes from two donors with CHM. Eyes were fixed and cryopreserved or the retina and choroid/RPE were processed as flatmounts with a small piece cut for transmission electron microscopy. A dense glial membrane, made up of vimentin and GFAP double-positive cells, occupied the subretinal space in the area of RPE and photoreceptor loss of both eyes. The membranes did not extend into the far periphery, where RPE and photoreceptors were viable. A glial membrane was also found on the vitreoretinal surface. Transmission electron microscopy analysis demonstrated prominence and disorganization of glial cells, which contained exosome-like vesicles. UEA lectin demonstrated complete absence of choriocapillaris in areas with RPE loss while some large choroidal vessels remained viable. In the far periphery, where the RPE monolayer was intact, choriocapillaris appeared normal. The extensive glial remodeling present in eyes with CHM should be taken into account when therapies such as stem cell replacement are considered as it could impede cells entering the retina. This gliosis would also need to be reversed to some extent for Müller cells to perform their normal homeostatic functions in the retina. Future studies investigating donor eyes as well as clinical imaging from carriers or those with earlier stages of CHM will prove valuable in understanding the glial changes, which could affect disease progression if they occur early. This would also provide insights into the progression of disease in the photoreceptor/RPE/choriocapillaris complex, which is crucial for identifying new treatments and finding the windows for treatment.

## Introduction

Choroideremia (CHM) is a rare recessive, X-linked disease affecting 1:50,000 individuals (1–3). Individuals with CHM experience varied degrees of pathology but most develop night blindness in adolescence. Vision loss begins in teenage years and progresses with patients having little or no visual function by age 60 (4).

The CHM gene encodes the Rab escort protein 1 (REP-1) (5). REP-1 is a membrane trafficking protein expressed in many tissues, including the retina and RPE. While there is evidence to suggest that REP-1 regulates retinal capillary networks and choriocapillaris (CC) as well as post-translational protein modification and intracellular molecular trafficking in RPE (5–7), we do not yet understand how defects in REP-1 cause the CHM clinical features. Although REP-1 is expressed in many cells in the retina as well, RPE are believed to be the first cell affected in CHM (7, 8). Recent advances in imaging technology, however, have created controversy as to where degeneration originates (8–13). Multi-modal imaging studies of CHM patients at varied disease stages revealed early RPE loss followed by photoreceptor outer segment (PR OS) loss and outer retinal tubulations (ORTs) in the affected border region (6, 14). ORTs were observed in all but the most severe cases (14). In addition to PR OS changes, recent imaging data has demonstrated thickening of the external limiting membrane (ELM) early in the disease process, before loss of RPE is noted, on optical coherence tomograophy (OCT) (11, 15–17). It has been speculated that this ELM thickening results from Müller cell remodeling in response to PR changes or stress signals, which occur prior to clinically observed changes.

Although glial remodeling has been demonstrated in other retinal degenerations (18–22) and was suggested in one electron microscopy study of a CHM donor eye (23), it has not been fully investigated in CHM. Similarly, while choroidal changes have been observed clinically, limited histopathologic assessments of choroidal loss have been done. In the present study, we investigated, using immunohistochemistry and transmission electron microscopy (TEM), glial remodeling and activation as well as choroidal changes and their association with RPE loss in the eyes from two patients with CHM.

## Methods

### Donor information

Donor 1 was an 81-year old Caucasian male who died of a myocardial infarction. In addition to CHM, this donor suffered from Parkinson’s disease and dementia. Donor 2 was a 74-year old Caucasian male who died of a heart attack. No other major medical history was noted for this donor. Although these donors had confirmed diagnosis of CHM, no genetic information was available.

### Tissue collection and handling

Eyes were received as direct donations through the Choroideremia Research Foundation. Eyes were harvested and shipped on wet ice by EverSight (Donor 1) and Lions Eye bank (Donor 2). Age-matched control tissue was received from the National Disease Research Interchange (NDRI) within 30 hrs post mortem. The eyes from Donor 1 were received 39 hrs post mortem. The eyes from donor 2 were fixed in 5% formalin at 24 hrs post mortem prior to shipping to the laboratory. Upon arrival in the laboratory, we removed the anterior chamber and, using a Stemi dissecting scope (Carl Zeiss Meditec, Inc), imaged the eyecups with the retina in place. From each donor, we cryopreserved one eye and processed the other for flatmount immunohistochemistry as described below. For control eyes, the retina was dissected from the choroid and fixed in 2% paraformaldehyde (prepared in 0.1 M cacodylate buffer; PFA). The RPE/choroid was incubated in 1% EDTA in dH2O for 2 hrs at room temperature before removing RPE with gentle pipetting and fixing the choroid in 2% PFA. All CHM eyes, however, contained dense scars which made it impossible to dissect the retina from the choroid except in the far periphery. The retina/choroid was fixed and stained together when they could not be separated. In parts that we could separate, the RPE was removed as described above before dissecting and fixing the choroid.

### Cryopreservation

One eye from each donor was cryopreserved according to our published method (19, 24, 25). Briefly, we fixed eyes for 2 hrs in 5% sucrose containing 2% PFA in PBS and then placed them in increased concentrations of sucrose before separating the posterior pole, inferior, nasal, temporal and superior pieces and freezing them in a 2:1 solution of 20% sucrose to optimal cutting temperature solution (TissueTek). Eight micron thick sagittal cross sections were cut on a Leica cryostat (Leica Biosystems).

### Transmission electron microscopy

A piece from each eye was fixed in 2.5% gluteraldehyde and 2% PFA (in 0.1M cacodylate buffer) for TEM. Tissue was processed as previously described (19, 26). Eye pieces were embedded so that cross sections could be cut. Images were collected using a Hitachi H7600 electron microscope.

### Immunohistochemistry

Immunohistochemistry on flatmounts or cryosections was performed as previously described (19, 27). For flatmounts, tissue was fixed overnight and then blocked in 5% goat serum prepared in 1XTBS with 1% Triton-X100 and 0.1% bovine serum albumin (TBST-BSA). Tissue was then incubated in primary antibodies (see **Table 1**; diluted in TBST-BSA) at 4°C for 72 hrs and then 48 hrs in secondary antibodies (diluted 1:200; see **Table 1**) along with Ulex Europaeus Agglutinin (UEA) lectin (1:100) in TBST. Antibody incubations were followed by three washes (20 min each) in TBST. Cryosections were blocked with 2% goat serum (prepared in TBST-BSA) for 20 min before incubating in primary antibody cocktail for 2 hrs and secondary antibody with DAPI (1:100) for 30 min. Secondary antibodies were prepared in TBS. TBS was used to wash sections between antibody incubations. We collected all images on a Zeiss 710 confocal microscope using Zen software. For mapping images, we obtained a tiled image (with 10% overlap) of each tissue piece at 1024x1024-pixel resolution as Z stacks using the 5x objective. Laser power, pinhole and gain were not changed between subjects for each marker analyzed.

**Table 1:** List of antibodies used.

Antibody	Source	Catalog #	Dilution
Rb-a-Glial fibrillary acidic protein (GFAP)	Dako	Z0334	1:200
Rb-a-vimentin	Abcam	AB45939	1:200
Ck-a- Glial fibrillary acidic protein (GFAP)	Millipore/Sigma	AB5541	1:500
Ck-a-vimentin	Millipore/Sigma	AB5733	1:500
Rb-a-Ionized calcium binding adaptor molecule 1 (IBA1)	Wako	019-19741	1:500
Ms-a-rhodopsin	Mybiosource	MBS803218	1:200
Rb-a-laminin	Millipore/Sigma	L-9393	1:200
UEA lectin	Genetex	GTX01512	1:100
Gt-a-Rabbit-cy3	Jackson Immunoresearch	111-165-003	1:200 flat 1:500 cyro
Gt-a-Rabbit AF647	Invitrogen	A21244	1:200 flat 1:500 cyro
Gt-a-Chicken-cy3	Jackson Immunoresearch	103-165-155	1:200 flat 1:500 cyro
Gt-a-Mouse-Cy3	Jackson Immunoresearch	103-165-155	1:200 flat 1:500 cyro
4′,6-diamidino-2-phenylindole (DAPI)	Invitrogen	D21490	1:1000

Table 1 antibodies used for flatmount or cryopreserved immunohistochemistry.

Rabbit (Rb), Chicken (Ck), Goat (Gt), Ulex Europaeus Agglutinin lectin (UEA lectin).

### Calculation of percent vascular area

The percent vascular area and CC diameters were calculated as previously described (28). Briefly, we exported maximum intensity projections of the 5x tiled images using Zen Software to full resolution Tiff images and opened them in Adobe Photoshop (CS6, Adobe Systems Inc.). Three regions (equivalent to 1mm^2^ each) were randomly selected from the posterior pole, equator and far periphery and pasted into a new document (27). After adjusting the threshold and levels, images were saved and opened in ImageJ. Images were converted to binary and noise was reduced (the same in each image) before calculating the black to white ratio, yielding the percent vascular area (29).

## Results

### Gross photography

**Figure 1:**
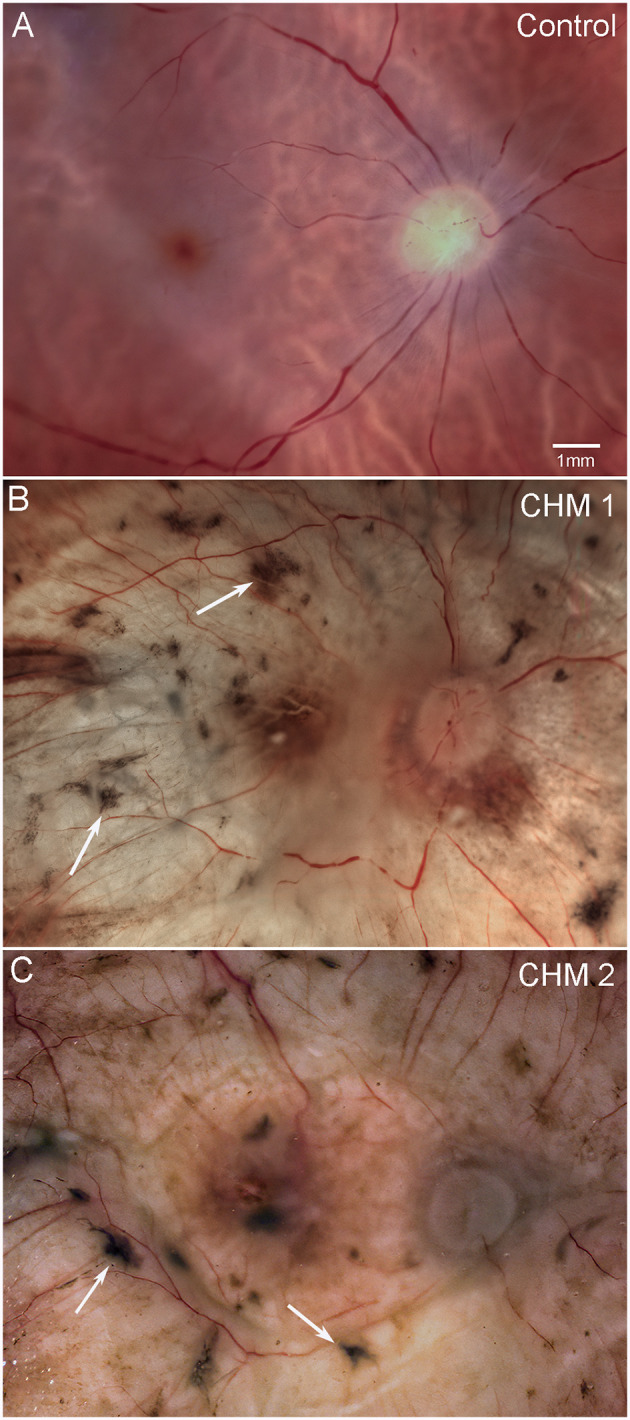
Gross photographs. Eyes from **(A)** age-matched control, **(B)** CHM Donor 1 and **(C)** CHM Donor 2 were imaged. Note the RPE atrophy, opaque membrane and bone spicules (arrows). Scale bar indicates 1mm and is the same in all images.

The control fundus shows a healthy retina with vascular arcades visible (**Figure 1A**). By contrast, both CHM retinas were cloudy and contained numerous pigmented spicules throughout (**Figures 1B, C**). Retinal vessels were visible but appeared abnormal and reduced in number. As mentioned above, we could not separate the retina from the choroid in either CHM eye due to a dense scar.

### Analyses of choroidal vasculature

**Figure 2:**
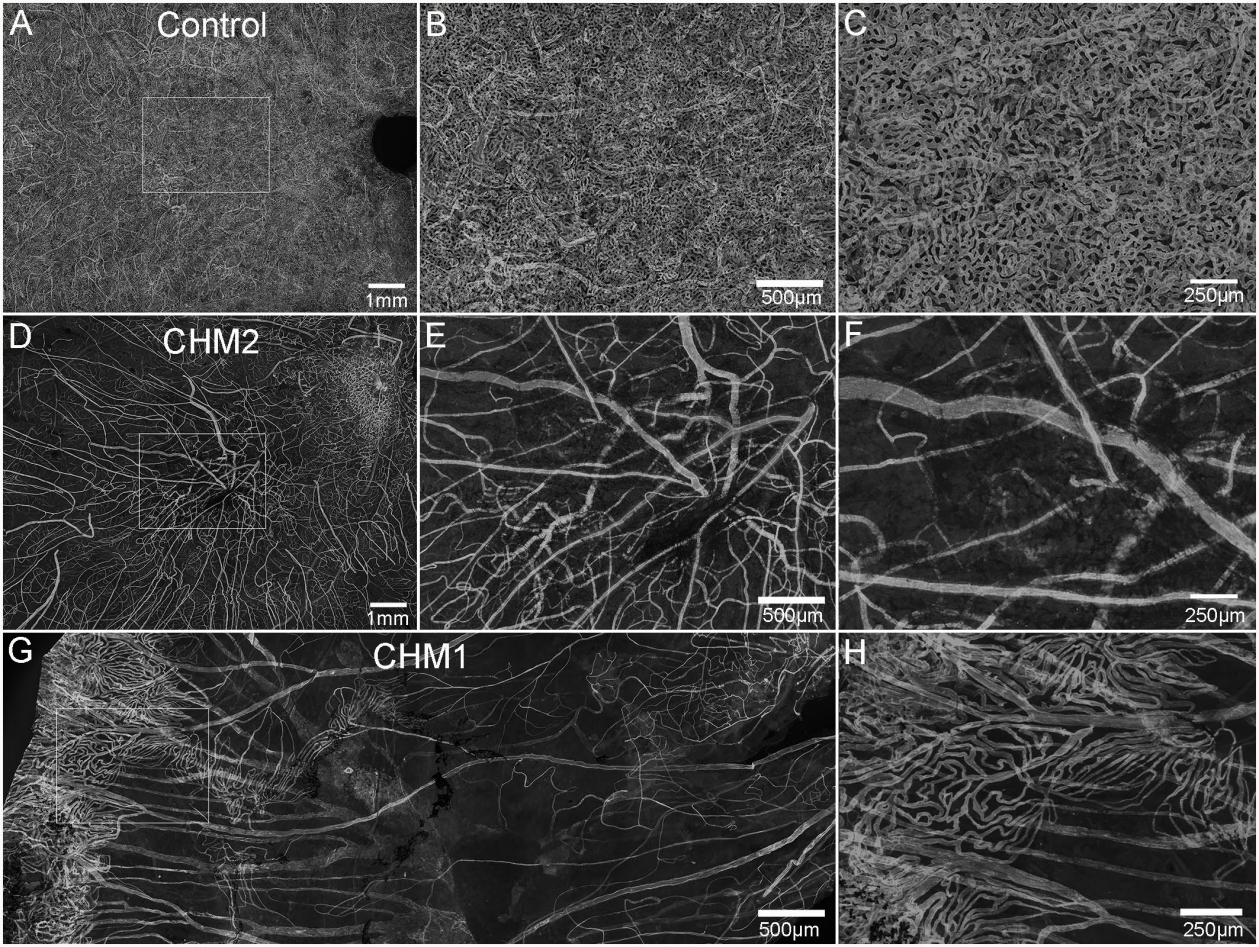
Choroidal vessels. Choroids from age-matched controls **(A–C)**, CHM Donor 2 **(D–F)** and CHM Donor 1 **(G, H)** were labelled with UEA lectin. The boxed areas in **A, D** are shown at high magnification in **B**, **C**, **E** and **F**. The boxed area in “**G**” is shown at higher magnification in **(H)**. Scale bars indicate: 1 mm **(A, D)**, 250 µm **(C, F, H)**, 500 µm **(B, E, G)**.

While the retina and choroid could not be separated, isolation of confocal planes allowed us to analyze the choroidal vessels. The control choroid showed organized, dense CC (**Figures 2A–C**). By contrast, only thinned large vessels remained throughout most of the CHM choroid (**Figures 2D–G**). The CC diameter in the posterior poles decreased from 18.3 (+/-2.1) µm in controls to 10.75 (+/-2.1) µm in the CHM eyes. Similarly, in the equator, CC diameter was reduced to 9.75 (+/- 2.1) µm in the CHM eyes whereas the control remained at 19.3 (+/-2.1) µm. In the far periphery, however, the CC diameter was similar to the control (Control: 24.5 +/-4 µm, CHM: 23.2 +/-3 µm). The percent vascular area in the posterior pole decreased from 78 (+/- 0.17) in controls to 17.1 (+/- 4.7) in CHM eyes. A similar pattern was observed in the mid-periphery where percent vascular area was 71.6 (+/-1.42) in controls compared to 12.7 (+/-3.6) in CHM eyes. In the periphery, however, the percent vascular area was fairly conserved (control: 62.5 +/- 4, CHM: 56.9 +/- 9.9). We also observed isolated islands with preserved CC in the CHM (**Figures 2G, H**). These regions appeared to correlate with intact RPE.

### Retinal vascular density

**Figure 3:**
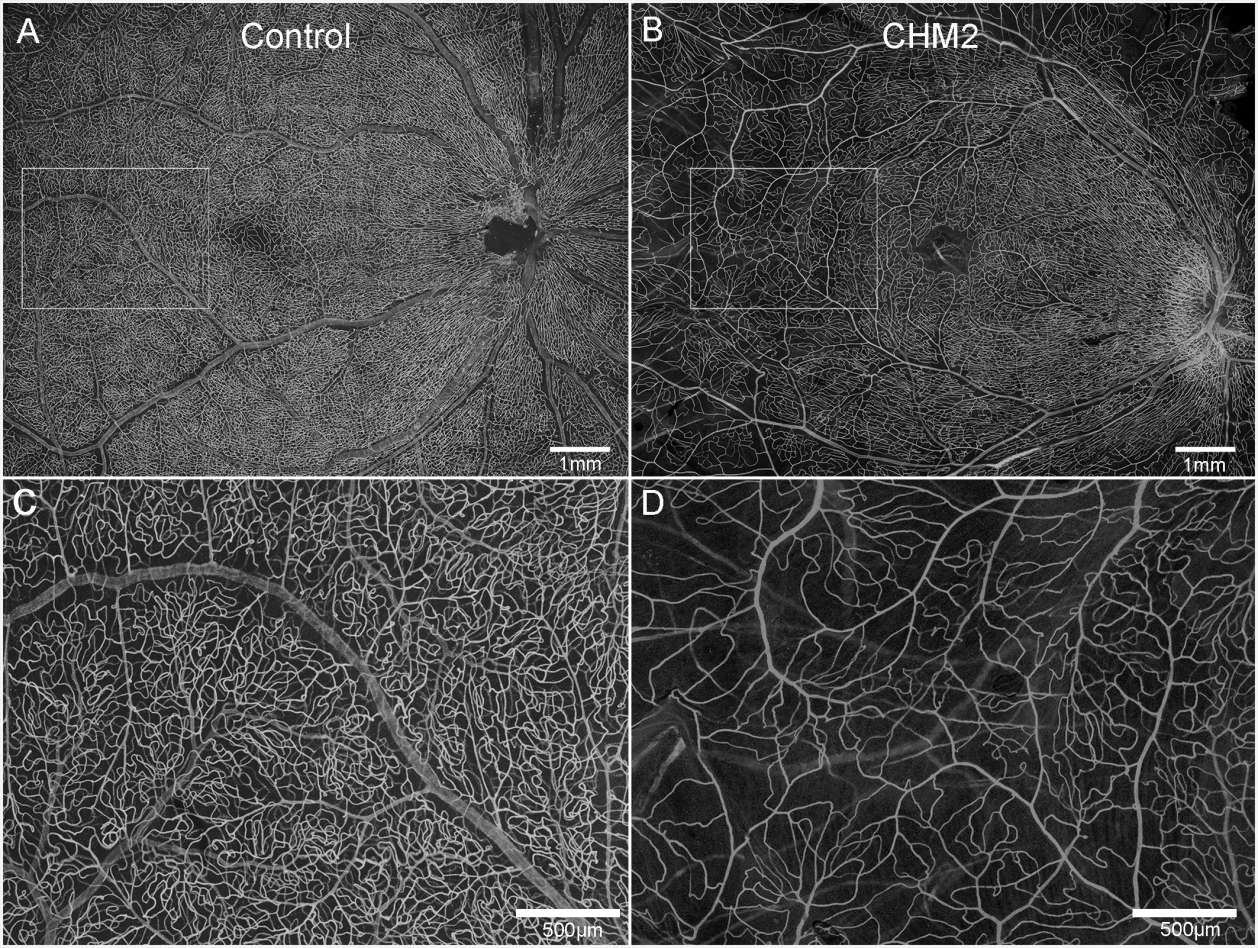
Retinal vasculature. UEA lectin staining of retinas from an age-matched control **(A)** and CHM Donor 2 **(B)**. Higher magnification of the boxed areas in **A** and **B** demonstrate the reduced vascular density in the CHM donor **(D)** compared to the control **(C)**. Scale bars indicate: 500 µm **(C, D)** and 1mm **(A, B)**.

The age-matched control retina demonstrated classic retinal vascular heirarchy with branches extending from the arcades and an avascular zone surrounding fovea (**Figures 3A, C**). Both CHM retinas, however, showed severely attenuated vasculature. In Donor 2's retina, the major arcades were intact but fewer branches were observed and the deeper secondary capillaries were largely missing (**Figures 3B, D**). The vessels were not as organized. Unfortunately, it was difficult to even discern the retinal vessels in Donor 1’s flatmount image.

### Glial remodeling and membrane formation

**Figure 4:**
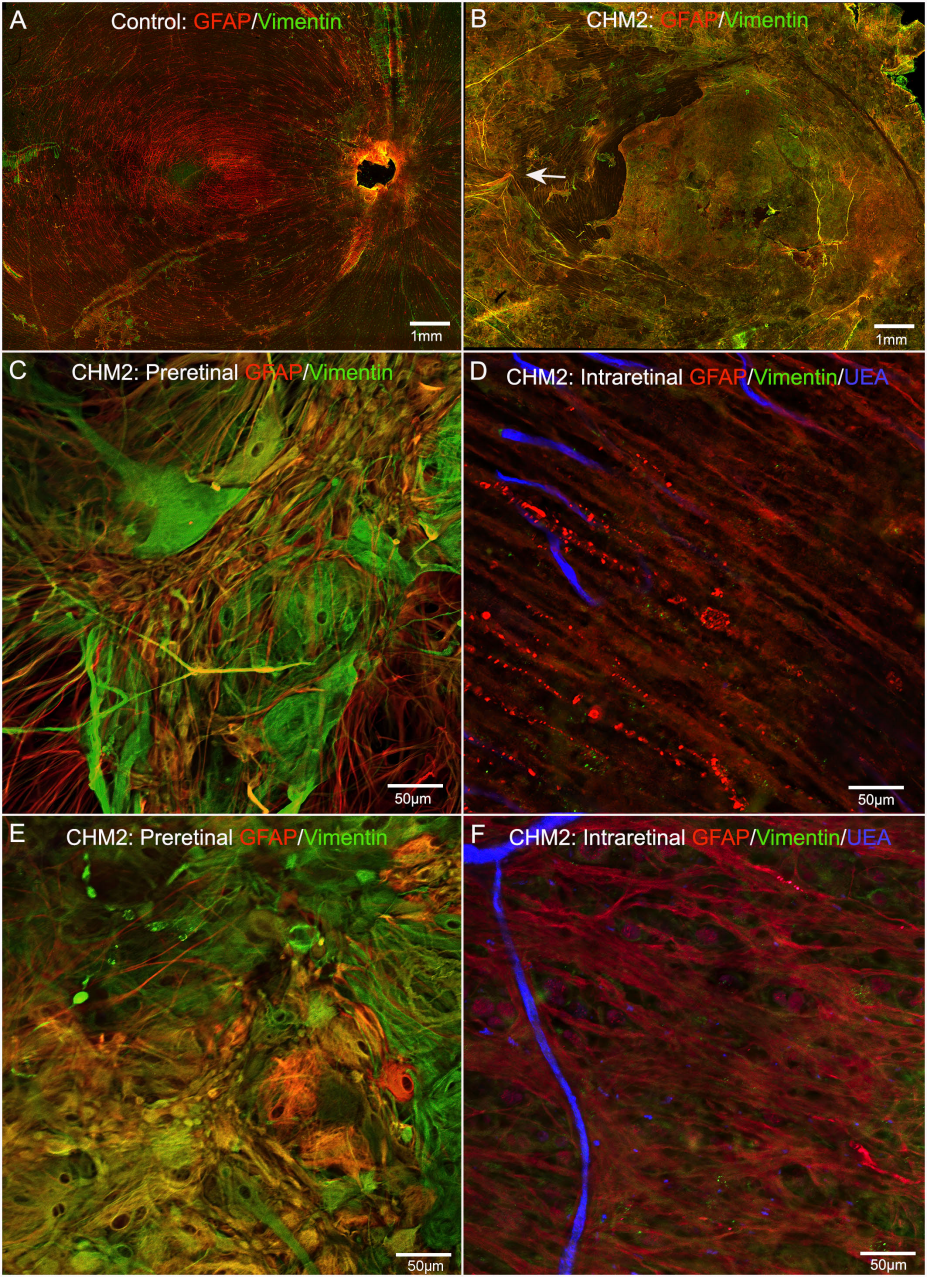
Retinal flatmounts imaged with the nerve fiber layer en face. Retinas from an age-matched control **(A)** and CHM Donor 2 **(B)** were stained with GFAP (red), vimentin (green) and UEA lectin (blue). The CHM retina is covered by a pre-retinal glial membrane positive for both GFAP and vimentin. The arrow in the CHM retina indicates “tented” preretinal glial cells causing traction on the vitreo-retinal surface. Higher magnification of the pre-retinal glial cells are shown in **(C, E)** with the retina in the focal plane below these images shown in **(D)**, **(F)** Scale bars indicate: 1 mm **(A, B)**, 50 µm **(C–F)**.

The control retina, stained with GFAP and vimentin demonstrated a uniform astrocyte template in a pattern similar to that of retinal vessels (**Figure 4A**). A few isolated glial cells were observed extending onto the vitreo-retinal surface. By sharp contrast, both CHM eyes had a dense membrane-like structure made of GFAP and vimentin-double positive cells as well as cells positive for only one of these proteins. This dense structure made visualizing the retinal arcades and astrocytes difficult at low magnification (**Figure 4B**). The complexity of this membrane and the retina below was better visualized at higher magnification (**Figures 4C, E**). As shown, numerous cells and processes occupied the vitreo-retinal surface of CHM eyes. These cells had different shapes and there was no organization to the structure they created. In one area, glial cells on the vitreo-retinal surface had a tented appearance, which indicates traction on the retina (**Figure 4B** arrow). When imaging into the deeper planes, a light staining of astrocytes was visible along with lectin staining for retinal vessels (**Figures 4D, F**). The GFAP staining of astrocytes did not reflect the morphology and well-defined pattern seen in the control. Similarly, the vessels were attenuated in the CHM eyes, as noted above. In the retina of Donor 1, examining sequential Z stack images revealed few deeper secondary retinal vessels and only isolated areas with GFAP-positive astrocytes. Rather, disorganized Müller cell processes were observed throughout the Z-stack, making it difficult to distinguish retinal layers (data not shown).

**Figure 5:**
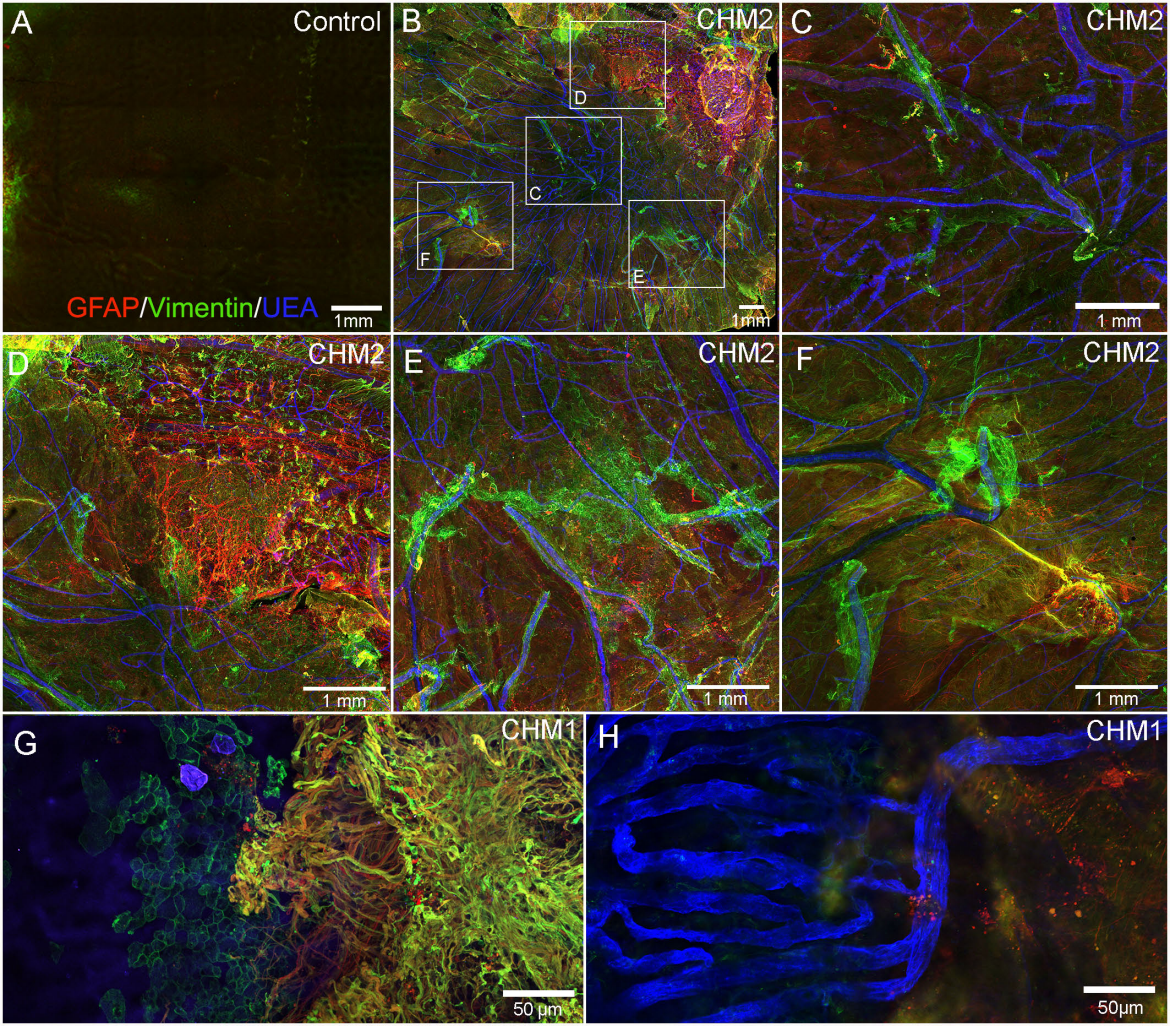
Retinal flatmounts imaged with the ELM layer en face. Retinas from an age-matched control **(A)** and CHM Donor 2 **(B)** were stained with GFAP (red), vimentin (green) and UEA lectin (blue) and low magnification tiled images were collected of the posterior pole. The areas boxed in **(B)** are shown at higher magnification to better visualize the glial membrane, composed of GFAP and vimentin-double positive cells **(C–F)**. High magnification of Donor 1’s peripheral retina imaged with the ELM en face shows the dense Müller cells in the subretinal space with a clear border but processes extend towards vimentin-positive RPE cells **(G)**. In the choroidal focal plane, CC vessels are observed in the far periphery, under remaining RPE cells **(H)**. Scale bars indicate: 1mm **(A–F)**, 50 µm **(G, H)**.

As mentioned above, the retinas of both CHM eyes were attached to the RPE/choroid by a thick scar-like structure. Imaging of retinal flatmounts with the ELM or, for the posterior poles, the choroid, en face demonstrated subretinal glial cells forming a second membrane-like structure similar to that seen on the vitreo-retinal surface. This was in sharp contrast to the non-descript staining of the control retina (**Figure 5A**). In CHM eyes, subretinal glial cells were positive for both GFAP and vimentin (**Figures 5B–G**). While these appear to be primarily Müller cell processes, the density of the membrane makes it difficult to rule out that cell bodies have also exited the retina. While the pre-retinal glial membrane covered the entire retina, the subretinal glia were confined to areas of RPE/CC atrophy. There was a very clear border in the far periphery where the glial membranes ended. Directly adjacent to this were intact RPE cells as well as a more complex CC than was observed in other areas (**Figures 5G, H**).

**Figure 6:**
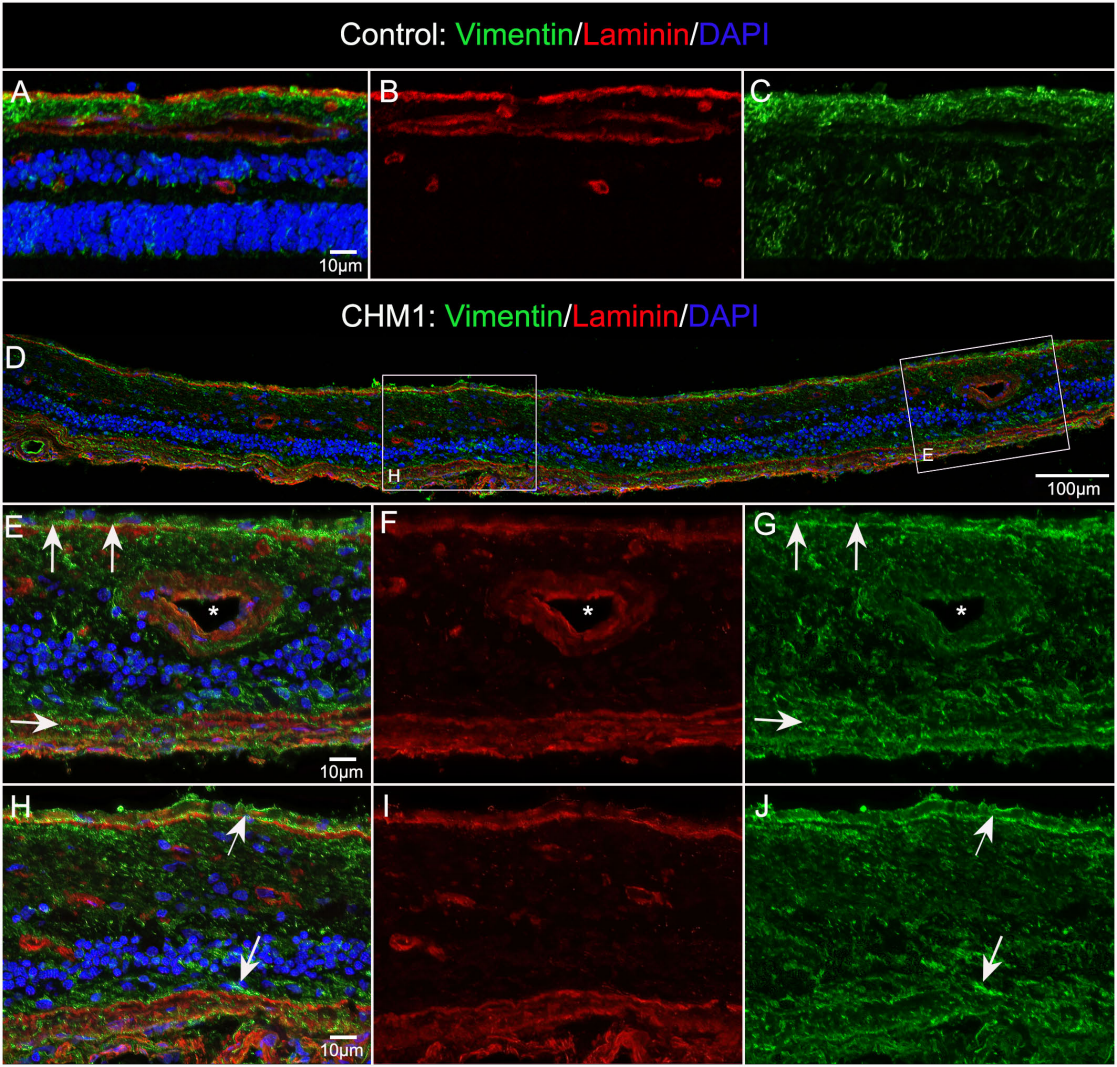
Müller cells viewed in cross section. Cryosections from age-matched control **(A–C)** and CHM Donor 1 **(D–J)** were stained with vimentin (green), laminin (red) and DAPI (blue). Higher magnifications **(E–J)** of the areas outlined with the corresponding box in “**D**”. Arrows in ,**(E & H)** indicate pre and subretinal glia. Asterisk indicates blood vessel lumen. Scale bars indicate: 10 µm **(A–C, E–J)**, 100 µm **(D)**.

Cross sections from the partner eye of each donor were also used to better understand glial structural changes. Sections from the posterior pole of Donor 1 stained with vimentin (Müller cells) and laminin (ILM and blood vessels), verified the presence of Müller cells on the vitreo-retinal surface (**Figures 6D–J**). These sections also confirmed that retinal lamination was lost. Müller cell processes and dislocated nuclei appeared to occupy much of the inner and outer retina. While the control Müller cell sections were fine and linear (**Figures 6A–C**), those from the CHM donor were thickened, askew and disorganized (**Figures 6E, G, H, J**). Sections from Donor 2 revealed similar findings (data not shown).

### Microglia were also associated with both pre and subretinal glial membranes

**Figure 7:**
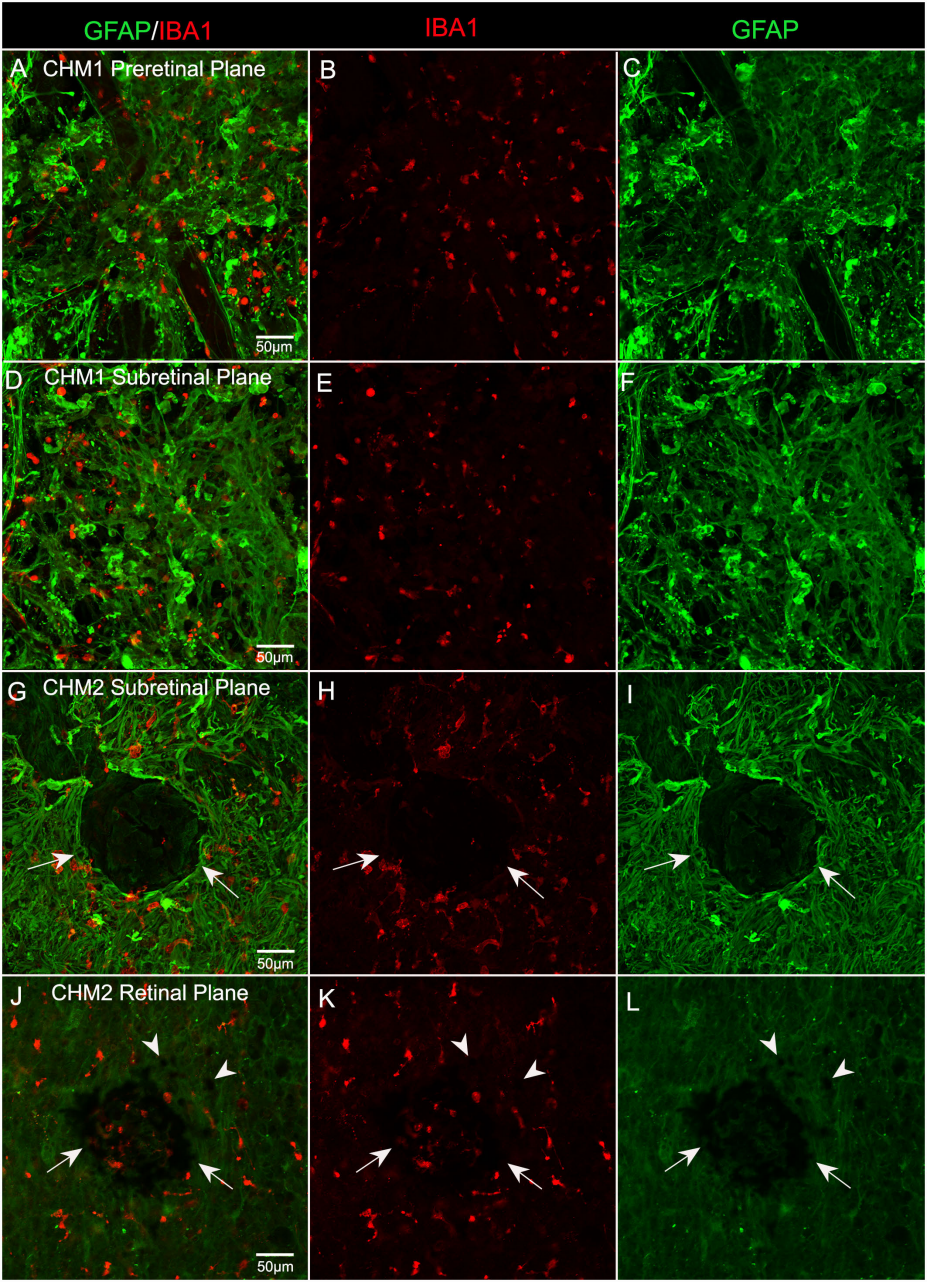
Microglia in CHM Donor flatmount retinas. CHM retinas were stained with GFAP (green) and IBA-1 (red). Staining with IBA-1 demonstrates the presence of microglia in both pre **(A–C)** and sub **(D–I)** retinal membranes. An unusual clump of pigmented cells or debris (arrows) are observed in the subretinal space surrounded by Müller cell processes and microglia in CHM Donor 2’s retina **(G–I)**. In the Z-stack focal plane within the retina from this same image **(J–L)**, pigmented cells are visible in this clump (arrows) as well as in the surrounding area (arrowheads). Scale bars indicate: 50 µm for all images.

In order to determine whether microglia were activated and associated with the pre-and subretinal glial membranes, we stained the inferior quadrant of both CHM retinas with GFAP and ionized calcium binding adaptor molecule 1 (IBA-1), a microglial marker. We observed numerous IBA-1 positive cells within both the pre- (**Figures 7A–C**) and subretinal glial membranes (**Figures 7D–F**). The microglia were rounded in appearance and lacked processes, demonstrating their presumed activation, in both membranes. In the eye from Donor 2, in the inferior retina, we observed a clump of pigmented cells, which may be migrated or detached RPE or bone spicules, surrounded by Müller cell processes and activated microglia (**Figures 7G–I**). In the Z-stack focal plane within the retina from this same image, this pigmented clump was still present as were pigmented cells in the the surrounding area (**Figures 7J–L**). These could be migrating RPE or bone spicules.

### Glial phagocytosis of outer segments

**Figure 8:**
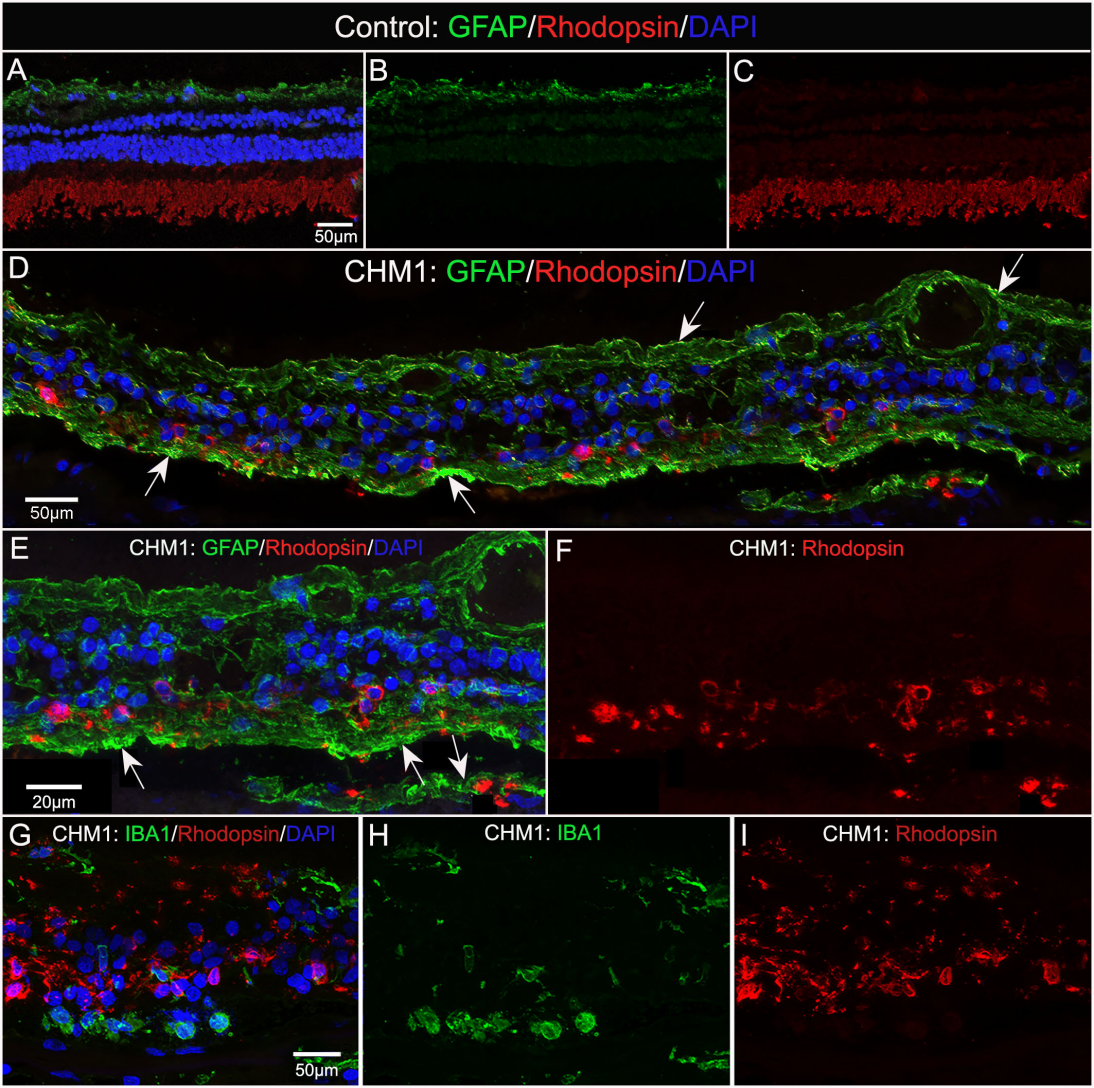
Cryosection analysis of glia and rhodopsin. Cryosections from an age-matched control **(A–C)** and CHM Donor 1 **(D–F)** were stained with GFAP (green) and rhodopsin (red) and DAPI (blue). In the control eye **(A–C)**, GFAP is confined to the astrocytes in the nerve fiber layer and rhodopsin is only observed in the photoreceptor segments. By sharp contrast, GFAP staining is observed throughout the CHM retina **(D–F)**, which was much thinner in appearance. Rhodopsin-positive debris is observed within Müller cell processes (arrows).Higher magnification **(E, F)** demonstrates the rhodopsin debris associated with GFAP-positive processes. IBA-1-positive cells **(G–I)** are not associated with rhodopsin debris. Scale bars: 50 µm **(A–D, G–I)**; 20 µm **(E, F)**.

We stained control and CHM donor cryosections with GFAP and rhodopsin to verify glial activation and determine whether any photoreceptor segments remained. For this analysis, we used sections from the mid-peripheral retina because this is where we observed some intact RPE in the gross photographs and flatmount analysis. GFAP staining in the control retina was confined to astrocytes in the nerve fiber layer (**Figures 8A, B**). Rhodopsin was confined to the photoreceptor segments (**Figures 8A, C**). Supporting our flatmount results, the CHM eyes contained GFAP^+^ processes throughout the extremely thin retina (**Figures 8D, E**). As mentioned above, the Müller cells were askew and appeared to be the dominant remaining retinal cell type. DAPI staining demonstrated complete lack of lamination in the retina. Despite the advanced disease state, rhodopsin-positive debris was observed (**Figures 8D, F**). This debris appeared to co-localize with Müller cell processes (**Figures 8D–F**). Therefore, we suspected the debris could represent phagocytosed photoreceptor outer segments. We stained adjacent sections with IBA-1 and rhodopsin to see if microglia were at least partly responsible for the rhodopsin. We did not observe co-localization between IBA-1 and rhodopsin (**Figures 8G–I**).

### Ultrastructure of glial cells

**Figure 9:**
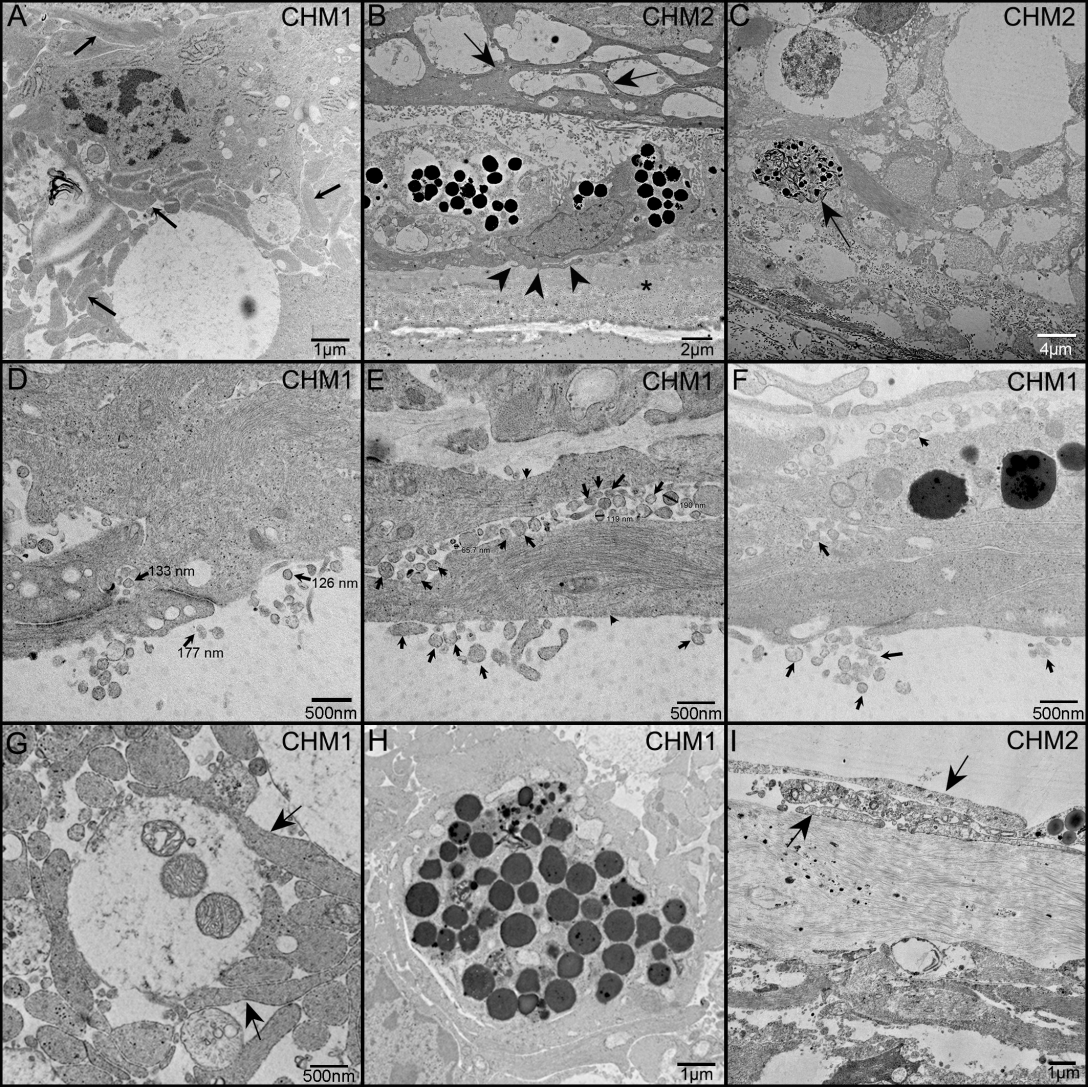
TEM analysis of the CHM Donor eyes. As observed in cross section, Müller cell processes (arrows) occupy much of the retinal space and have lost their linear morphology **(A)**. Müller cell processes are evident between Müller cells in the subretinal space (arrows) as well as beneath the RPE and extending towards Bruch’s membrane (arrowheads) **(B)**. Asterisk indicates Bruch’s membrane. Photoreceptor outer segment debris is observed in Müller cells **(C)**. Extracellular vesicles (arrows), the size of exosomes, are seen exiting Müller cells (arrowheads) and throughout the retina and subretinal space **(D–F)**. Tight junctions between Müller cell processes are also evident in **(D)**. Müller cell processes (arrows) ensheath possible photoreceptor outer segment fragments **(G)**. Müller cell processes surround a clump of pigmented material, possibly bone spicules **(H)**. Glial cells (arrows) are evident on the vitreal side of the missing or very thin ILM **(I)**. Scale bars indicate: 1 µm **(A, H, I)**, 2 µm **(B)**, 4 µm **(C)**, 500 nm **(D–G)**.

TEM analysis was used to examine the ultrastructure of the CHM eyes. This analysis confirmed that Müller cell processes were prominent in CHM retinas and had lost their linear morphology (**Figure 9A**, arrows). We also observed the Müller cell processes occupying the subretinal space and extending into Bruch’s membrane (**Figure 9B**). Importantly, Müller cell tight junctions were observed in the subretinal space (**Figure 9D**). We also observed photoreceptor outer segment debris within Müller cells (**Figures 9C, G**), supporting our immunohistochemical observations of rhodopsin within glial cells (**Figures 8D–F**). Extracellular vesicles, the size of exosomes, were also noted presumably exiting from Müller cells throughout the retina as well as in the subretinal space (**Figures 9D–F**). We also observed a condensed area of bone spicules in the inner retina surrounded by Müller cell processes (**Figure 9H**). This was reminiscent of the structure reported in Donor 1’s flatmount when stained with IBA1 and GFAP (**Figure 7G–I**). The pre-retinal glial membrane was also verified by TEM (**Figure 9I**). The ILM was absent or very thin in some areas and Müller cells did not have well formed endfeet.

## Discussion

In this report, we have provided histopathologic and immunohistochemical evidence of extensive Müller cell activation and remodeling in eyes from two CHM patients. Moreover, choroidal and retinal vascular densities were severely reduced in both the posterior and equatorial regions of these eyes. Since the donors reported herein were in their seventh and eighth decades, it is difficult to discern much regarding the pathological process from their eyes. Identifying the pathologic changes, even in advanced stages, however, can help direct how and when we treat CHM patients.

The reduced retinal vasculature is not surprising given the PR loss and retinal thinning in the CHM eyes. Retinal vascular density thinning has also been reported with optical coherence tomography angiography (OCT-A) (16). The reduction could be a biological response to retinal neuronal death. The correlation between retinal vascular density and thinning is supported by the observation that Donor 2, who was 7 yrs younger, had more preserved retinal vessels than Donor 1 and his retinal thinning was not as severe. It is important to note, however, that reduced retinal thickness and vasculature have recently been observed in patients with Parkinson’s disease (30). Therefore, Donor 1’s Parkinson’s diagnosis could have been a confounding factor in his retinal thinning. A recent study used OCT and OCTA to correlate retinal and choroidal vascular density with changes of thickness in retinal layers of CHM patients in their 30s (16). Arrigo and colleagues observed that the ganglion cell layer, outer nuclear layer, RPE and choroid were thinner in the atrophic area compared to healthy area. By contrast, the inner nuclear and inner plexiform layers were thinner in both the atrophic and preserved areas. The deep capillary plexus also had a lower vascular density compared to normal patients in both the atrophic and healthy areas. The CC, however, was only reduced in the atrophic area. Based on these observations, the authors speculated that inner retinal changes, separate from RPE loss, could cause reduced retinal vasculature (16). Despite the retinal vascular loss, the persistence of some vessels, particularly in the inner retina, suggests there is structural support for new cells, such as stem cell-based replacement therapies.

As expected, the choroid was severely attenuated in CHM eyes. While only large blood vessels survived throughout most of the choroid, there were CC vessels present in the far periphery of both CHM eyes. Isolated CC islands were also observed in other areas. Although we only observed areas with conserved CC with surviving RPE cells, the data obtained herein does not provide evidence regarding whether the RPE or CC is affected first. Current studies in our laboratory are investigating the state of the surviving RPE cells, which may provide clues regarding disease pathology. Although many believe RPE cells are the first to be affected in CHM, the contribution of choroidal changes to the pathological process cannot be ruled out. A study by Cameron and colleagues reported, using electron microscopy, increased production of basement membrane by choroidal endothelial cells in a 66 yr old CHM donor eye (23). These authors suggested that changes in the choroidal vessel walls led to reduced lumen size and noted similar changes in the iris vessels. In the iris, vascular changes preceded areas with pigment epithelial defects. These authors speculated that defects in choroidal endothelial cells could contribute to CHM pathology. While we did not observe these changes in the choroidal vessel walls, this may be due to the more advanced stages of CHM donor eyes in this study. Further studies on choroidal changes in CHM, both in tissue and using cell culture techniques, could help identify therapeutic targets.

Based on our observations that both RPE and CC were present in the peripheral choroid and the fact that no areas were observed with only RPE or CC, it seems likely that they are lost in close succession. This idea is supported by the fact that recent imaging studies have found differing results regarding what cell is affected first (11, 17, 31–33). It is also possible that multiple cell types contribute to the pathology of CHM. Therefore, loss of CC should be an important consideration when deciding on treatment options. For example, there may be a brief window after RPE loss before subsequent CC degeneration occurs when RPE replacement therapy would be most beneficial.

We also investigated the structure of the retina-choroidal scar, which prevented removal of the retina in CHM eyes. This subretinal membrane-like structure was complex and appeared to contain multiple layers of Müller cell processes and cells. IBA1-positive microglia and macrophages were also observed within this glial structure but were not as prominent as Müller cells. The Müller processes extended through Bruch’s membrane and into the choroid where they ensheathed choroidal vessels, as shown in flatmount analysis. This observation confirms an earlier electron microscopy study which demonstrated glial processes on the choroidal side of Bruch’s membrane of a 66 yr old CHM donor (23). While the earlier report demonstrated glial cells had their polarity disrupted with endfeet on the choroidal side of Bruch’s membrane, we did not observe this phenomenon in our analysis. This could be due to the advanced stages of our donor eyes and the increased number of glial processes present in the subretinal space. Given the disorganization of the Müller glia, it would not be surprising if some had altered polarity. We did observe a lack of normal Müller cell endfeet at the ILM. Similar glial remodeling and membrane formation has been observed in areas of atrophy in geographic atrophy (18), retinitis pigmentosa (personal observation, unpublished), retinal detachment (34) and retinal degeneration (35). It is believed that Müller cells extend their processes in response to the loss of their binding partners at the ELM, the photoreceptors, in an attempt to create a new ELM with other Müller cells. Indeed, we did observe tight junctions between Müller cells in the subretinal space. This could be beneficial, protecting the retina from substances in the subretinal space. Alternatively, such a membrane could create a barrier that would hinder treatments from reaching the retina. In particular, transplanted photoreceptor or RPE cells would be blocked from their target location by Müller cell processes. Moreover, gene therapy would also be difficult because the glial scar would hinder the local detachment of retina required for subretinal injections. It is also not known how these Müller cells interact with native RPE or transplanted photoreceptor precursors in cell replacement therapy.

We have yet to determine whether Müller cells making up subretinal membranes maintain their normal functions or if they transition to more fibrotic cells. Similarly, the homeostatic function of Müller cells within the retina, which are disorganized and have lost their linear morphology, may also be affected in CHM eyes. While we cannot determine how early in the disease process Müller cell changes occur, an OCT study demonstrated retinal thickening and changes at the ELM before photoreceptor or RPE loss (11, 17). These authors speculated that the changes were due to remodeling of Müller cells, an idea supported by our data. Since it was not possible with the tissue in the current study, future studies will investigate how this remodeling affects Müller cell function. If Müller cell function is disrupted, this could exacerbate photoreceptor loss. The OCT/OCTA study mentioned above by Arrigo and colleagues points towards Müller cell involvement early in the disease process which could occur along with RPE loss (15). Müller cell changes within the retina are also important to consider with stem cell-based therapy. One important consideration is whether Müller cells will return to normal and be able to support transplanted cells.

The pre-retinal glial membrane contained both Müller cells (GFAP and vimentin-positive) and astrocytes (positive for GFAP only). Similar glial membranes were reported in CHM eyes with electron microscopy (23). The tenting of glia observed Donor 2’s retina indicates the possibility that the pre-retinal membrane could exert traction on the retina, leading to retinal detachment or macular holes. Indeed, macular holes have been reported in CHM patients (36). In addition, this membrane could impact the ability of intravitreally injected treatments to reach the outer retina. Furthermore, activated Müller cells release inflammatory cytokines and could exacerbate the inflammatory response that can be associated with gene therapy.

The presence of exosomes in and around Müller cells suggests that these cells use exosomes as a means of communication. To our knowledge, Müller cell exosomes have not yet been reported in human donor eyes. In addition, we observed Müller cells phagocytosing photoreceptor debris in the peripheral retina of Donor 1. This observation was surprising given the advanced disease stage but suggests that peripheral photoreceptors survived until fairly recently.

We acknowledge that the long post-mortem time for the CHM eyes is a shortfall of this study. We feel confident, however, in the results presented because the antibodies we are using have been shown to withstand long post mortem times without affecting efficacy in our hands and by others. Also, we are not comparing expression levels except for in the case of GFAP where expression is dramatically increased in the Müller cells of the CHM eyes. The excessive remodeling of Müller cells or loss of choroidal vessels would not occur due to increased post mortem time. The rarity of these CHM eyes makes it worth reporting the results despite the post-mortem delay.

In conclusion, our findings indicate that for any cell or gene-based therapy to successfully treat CHM, they must be employed early before there is extensive glial scarring and choroidal vascular loss. It is important that, as imaging capabilities improve, clinicians are aware of potential glial membrane formation at either the ILM or the subretinal space as this may determine the best mode for treatment delivery. It is critical that we understand the pathological changes in all cells involved so that we can identify novel therapeutic targets that may slow or even prevent CHM disease progression. Clearly, treatments for CHM will evolve as our understanding of the disease process improves.

## Data availability statement

The original contributions presented in the study are included in the article/supplementary material. Further inquiries can be directed to the corresponding author.

## Ethics statement

Written informed consent was obtained from the individual(s) for the publication of any potentially identifiable images or data included in this article.

## Author contributions

GL organized eye donations and, along with ME, designed experiments and interpreted data. ME performed flatmount immunohistochemistry, confocal microscopy, data analysis, and wrote manuscript. DM assisted with confocal imaging, figure preparation and writing of the manuscript. IB collected gross photographs, prepared eyes for histological assessment (flatmounts or cryopreservation) and cut cryosections. RD and KC performed immunohistochemistry studies on cryosections and assisted with confocal imaging. RG prepared samples for TEM and conducted all TEM analysis. All authors reviewed and edited the manuscript. All authors contributed to the article and approved the submitted version.

## Funding

Choroideremia Research Foundation (GL), BrightFocus Foundation (MME), NEI/NIH EY01651 (GL & ME), EY031044 (ME), EY001765 (Wilmer Eye Institute) and Research to Prevent Blindness Unrestricted Grant (Wilmer Eye Institute).

## Acknowledgments

This manuscript is dedicated to our late mentor, friend and collaborator, GL, as it would not have been possible without his foresight, dedication and wisdom. We cannot express enough our gratitude to the donors and their families for their donation to science.

## Conflict of interest

The authors declare that the research was conducted in the absence of any commercial or financial relationships that could be construed as a potential conflict of interest.

## Publisher’s note

All claims expressed in this article are solely those of the authors and do not necessarily represent those of their affiliated organizations, or those of the publisher, the editors and the reviewers. Any product that may be evaluated in this article, or claim that may be made by its manufacturer, is not guaranteed or endorsed by the publisher.
